# Efficacy and safety of Shenyankangfu tablets for primary glomerulonephritis: study protocol for a randomized controlled trial

**DOI:** 10.1186/1745-6215-15-479

**Published:** 2014-12-05

**Authors:** Jia Kou, Jie Wu, Hong-tao Yang, Ya-ni He, Jing-ai Fang, Yue-yi Deng, Yuan-sheng Xie, Li-fang Nie, Hong-li Lin, Guang-yan Cai, Xiang-mei Chen

**Affiliations:** Department of Nephrology, Chinese PLA General Hospital, Chinese PLA Institute of Nephrology, State Key Laboratory of Kidney Diseases, National Clinical Research Center of Kidney Diseases, 28 Fuxing Road, Haidian District, Beijing, 100853 China; Department of Nephrology, The First Affiliated Hospital of Tianjin University of TCM, 314 West Anshan Road, Nankai District, Tianjin, 300193 China; Department of Nephrology, Daping Hospital, The Third Military Medical University, 10 Changjiangzhilu Daping, Yuzhong District, Chongqing, 400042 China; Department of Nephrology, First Hospital of Shanxi Medical University, 85 liberation Road, Taiyuan, Shanxi Province 030001 China; Department of Nephrology, Longhua Hospital of Shanghai University of TCM, 725 South Wanping Road, Shanghai, 200032 China; Department of Nephrology, Xiyuan Hospital CACMS, 1 Xiyuan Playground, Haidian District, Beijing, 100091 China; Department of Nephrology, The First Affiliated Hospital of Dalian Medical University, 222 Zhongshan Road, Zhongshan District, Dalian, Liaoning Province 116011 China

**Keywords:** Glomerulonephritis, Proteinuria, Shenyankangfu tablets, Losartan, Efficacy, Safety, Randomized controlled trial, Double-blind

## Abstract

**Background:**

Chronic kidney disease is a common disease. Most chronic kidney diseases evolve from primary glomerulonephritis. Proteinuria is an independent risk factor for the progression of chronic kidney disease. The general consensus is that therapy administered to decrease proteinuria should include steroids and/or immunosuppressants, angiotensin-converting enzyme inhibitors, and angiotensin II receptor blockers. However, the side effects of, and adverse reactions to, these agents reduce the benefits to patients. In addition, the cost of these drugs is relatively high. Therefore, identification of inexpensive and effective drugs to decrease proteinuria is urgently needed. Shenyankangfu tablets have been a widely applied Chinese patent medicine for many years to decrease proteinuria. However, there is a lack of research-derived data regarding the clinical use. Therefore, we designed the present randomized controlled clinical trial to compare the efficacy and safety of Shenyankangfu tablets versus losartan potassium for control of proteinuria in patients with primary glomerulonephritis.

**Methods/design:**

This study will be a multicenter, prospective, double-blind, double-dummy, randomized controlled clinical trial. We will enroll 720 patients diagnosed with primary glomerulonephritis. The eligible patients will be randomly divided into the following groups at a 1:1:1:1:1 ratio: Shenyankangfu tablets group, losartan potassium 50 mg group, losartan potassium 100 mg group, Shenyankangfu tablets + losartan potassium 50 mg group, and Shenyankangfu tablets + losartan potassium 100 mg group. All groups will be followed up for 48 weeks; follow-up visits will be performed, at weeks 0, 4, 8, 12, 24, 36, and 48. The primary efficacy outcome will be the post-treatment change in the 24-hour proteinuria level, and the secondary efficacy outcomes will be the post-treatment changes in the serum creatinine level, estimated glomerular filtration rate, traditional Chinese medicine syndrome score, and serum albumin level.

**Discussion:**

The results of this trial will provide solid data for use in evidence-based medicine with respect to the efficacy and safety of Shenyankangfu tablets for control of proteinuria in patients with primary glomerulonephritis compared to those of losartan potassium. Moreover, we infer that therapy comprising Shenyankangfu tablets + losartan potassium can decrease proteinuria to a larger extent than Shenyankangfu tablets or losartan potassium can alone.

**Trial registration:**

This trial was registered on 12 February 2014 at ClinicalTrials.gov (ID number NCT02063100).

**Electronic supplementary material:**

The online version of this article (doi:10.1186/1745-6215-15-479) contains supplementary material, which is available to authorized users.

## Background

Chronic kidney disease (CKD) is a common disease, and its major component is primary glomerulonephritis. CKD is prevalent in developed countries [1, 2], and it is estimated that 11.5% of adults in the United States have CKD [3]. The overall prevalence rate of CKD in China is 10.8% (10.2 to 11.3%) [4], and the disease has become an important public health problem in this country. CKD significantly promotes the occurrence of end-stage renal disease worldwide [5, 6]. Peterson and colleagues [7] initially identified proteinuria as an independent risk factor for nondiabetic CKD. In addition, many studies have demonstrated that proteinuria is a risk factor for all-cause mortality, cardiovascular mortality, CKD progression, and renal failure [8–12]. Furthermore, reduction of proteinuria has been shown to slow the progression of CKD [13–17].

In Western medicine, steroids and/or immunosuppressants are used to decrease proteinuria [18]. However, the long-term use of steroids and/or immunosuppressants is associated with a series of serious side effects including muscle atrophy, centripetal obesity, hypertension, high cholesterol, and development or aggravation of infections. Widely used therapies for reducing proteinuria also include angiotensin-converting enzyme inhibitors (ACEI) and angiotensin II receptor blockers (ARB) [18, 19]. Data from evidence-based medicine have proven that ACEI and ARB play important roles in delaying the progression of CKD and providing an early renoprotective effect by controlling blood pressure and proteinuria [20–23]. However, research has shown that long-term medication may lead to a number of adverse reactions such as coughing, hypotension, hyperkalemia, exacerbated kidney function, and negative effects on the fetus [24]. The costs of these drugs are relatively high and represent an economic burden for most Chinese patients. Therefore, identification of inexpensive and effective drugs that decrease proteinuria is urgently needed.

Shenyankangfu tablets (SYKFT) are a Chinese patent medicine used to treat patients with chronic glomerulonephritis, proteinuria, hematuria, and Qi-Yin deficiency syndrome according to the traditional Chinese medicine diagnostic criteria. The Chinese term *Shenyankangfu* refers to the cure of glomerulonephritis and promotion of patients’ rehabilitation. The clinical application of SYKFT began in 1994, and this medication has since been widely applied throughout China. It is inexpensive and has demonstrated excellent efficacy and safety in decreasing proteinuria without toxicity or side effects. Despite the above-mentioned advantages of SYKFT, its application is limited due to a lack of evidence derived from randomized controlled clinical trials. Therefore, we have designed a multicenter, prospective, double-blind, double-dummy, randomized controlled clinical trial to compare the efficacy and safety of SYKFT versus losartan potassium for control of proteinuria in patients with primary glomerulonephritis.

Losartan potassium is one of the earliest ARB used to treat CKD based on large amounts of research-derived data regarding its ability to decrease proteinuria and exert renoprotective effects [25–31]. Therefore, it will be used as the control medication in the present trial.

## Methods/design

### Trial design

**Figure 1:**
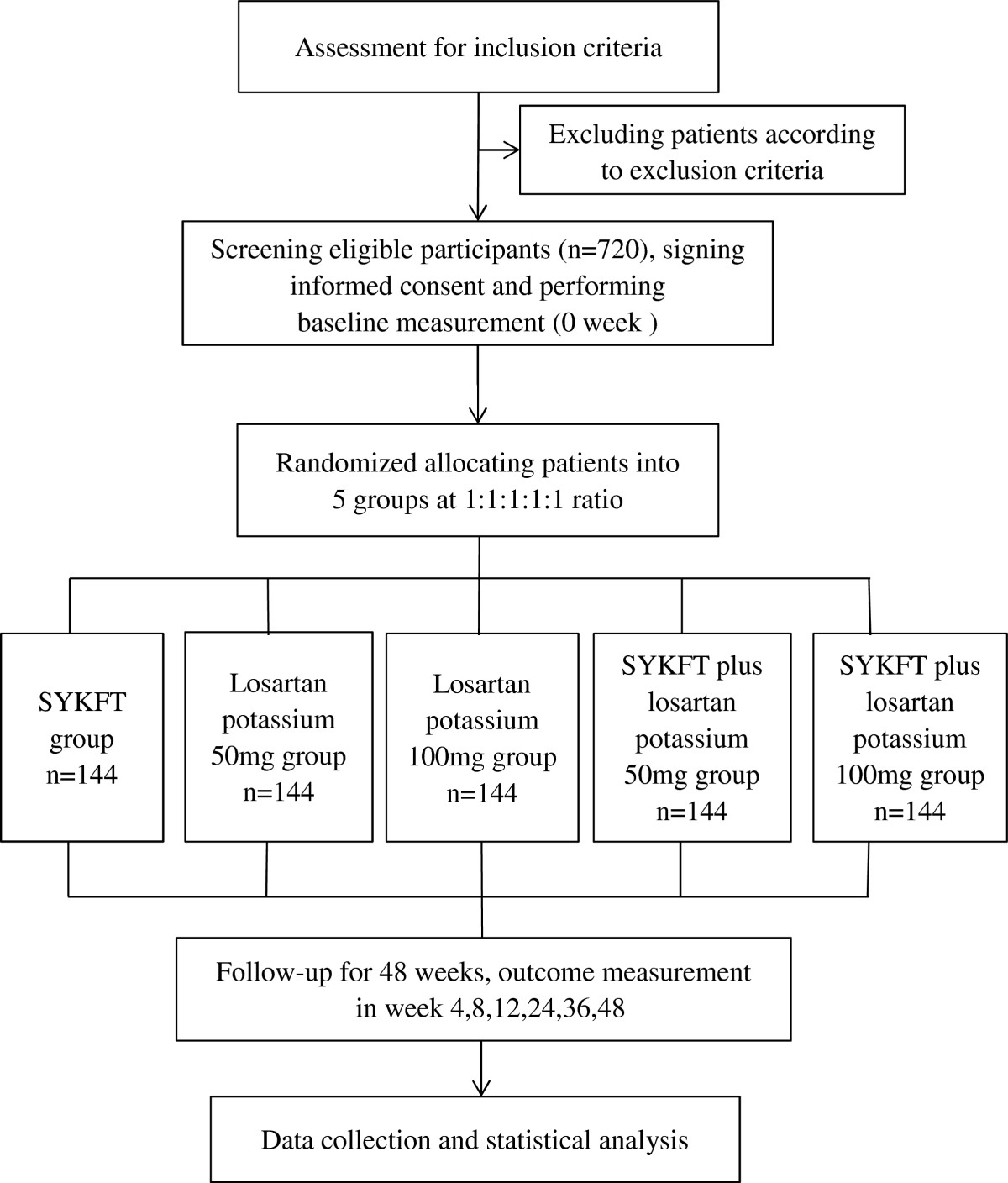
**Trial flow diagram.** SYKFT, Shenyankangfu Tablets.

The study will be a multicenter, prospective, double-blind, double-dummy, randomized controlled clinical trial. The protocol has been repeatedly discussed among experts, and all investigators have agreed on the finalized protocol. The study will be performed at various sites among 19 provinces and municipalities. In total, 720 eligible patients will be recruited and randomly assigned to five groups at a 1:1:1:1:1 ratio. A 48-week follow-up will be conducted; outcome measurements will be performed at weeks 0, 4, 8, 12, 24, 36, and 48 (Figure 1). The protocol has been approved by the institutional review board and/or independent ethics committee of each study site. More detailed information about the ethical approval is listed in Additional file 1. All participants will sign written informed consent forms before undergoing any study-related procedures. The participants will have sufficient time to read the informed consent form and ask about the details of the trial before providing written consent. The study will be conducted according to the established study protocol, the principles of the current version of the Declaration of Helsinki [32], the International Conference on Harmonization guidelines for Good Clinical Practice, and the Good Clinical Practice guidelines of the China Food and Drug Administration [33, 34]. The trial has been registered at ClinicalTrials.gov (ID number NCT02063100). The entire study will be monitored by Tianjin Taicheng Yaozhong Biomedical Technology Co. Ltd, a contract research organization.

### Participants

We propose to enroll 720 participants, mainly from the kidney disease outpatient and inpatient departments. The inclusion criteria are a diagnosis of primary glomerulonephritis, aged 18 to 70 years, blood pressure ≤140/90 mmHg, estimated glomerular filtration rate ≥45 mL/min/1.73 m^2^, 24-hour proteinuria level of 0.5 to 3.0 g, traditional Chinese medicine syndrome conforming to Qi-Yin deficiency, and agreement to participate by the patient or their guardians with written informed consent.

The exclusion criteria are: secondary nephropathy; therapy with glucocorticoids, immunosuppressants, or *Tripterygium* drugs in the last 12 months; therapy with other Chinese patent medicines or decoctions that can reduce proteinuria in the last 2 weeks; therapy with renin-angiotensin system blockers in the last 4 weeks; severe primary disease of the heart, brain, liver, or hematopoietic system, or other serious diseases that can affect the patient’s life; pregnancy or lactation; allergic predisposition or known allergy to the drug composition; blood pressure <90/60 mmHg; unilateral or bilateral renal artery stenosis; mental disorders and poor compliance; suspected or confirmed history of alcohol or drug abuse; and participation in another clinical study at the same period (Table 1).

**Table 1:** **Inclusion and exclusion criteria**

Inclusion criteria	Exclusion criteria
(1) Diagnosis of primary glomerulonephritis	(1) Secondary nephropathy
(2) Aged 18 to 70 years	(2) Taken glucocorticoid, immunosuppressants or *Tripterygium* drugs in the last 12 months
(3) Blood pressure ≤140/90 mmHg	(3) Therapy with other Chinese patent medicines or decoctions that can reduce proteinuria in the last 2 weeks
(4) Estimated glomerular filtration rate ≥45 mL/min/1.73 m^2^	(4) Therapy with renin-angiotensin system blockers in the last 4 weeks
(5) 24-hour proteinuria level 0.5 to 3.0 g	(5) Severe primary disease of the heart, brain, liver, or hematopoietic system, or other serious diseases that can affect the patient’s life
(6) Traditional Chinese medicine syndrome conforming to Qi-Yin deficiency	(6) Pregnancy or lactation
(7) Agreement to participate by the patient or their guardians with written informed consent	(7) Allergic predisposition or known allergy to the drug composition
	(8) Blood pressure <90/60 mmHg
	(9) Unilateral or bilateral renal artery stenosis
	(10) Mental disorders and poor compliance
	(11) Suspected or confirmed history of alcohol or drug abuse
	(12) Participation in another clinical study at the same period

According to the Guidelines for Clinical Research of Chinese Medicine (New Drug) [35], for a patient’s traditional Chinese medicine syndrome conforming to the Qi-Yin deficiency syndrome established in the inclusion criteria, the patient must have at least two of the three primary symptoms and at least one of the five secondary symptoms, and have relevant tongue and pulse conditions. The three primary symptoms are weak mental state and physical fatigue, thirst and dry mouth and throat, and waist soreness with weak knees. The five secondary symptoms are feverish palms and soles, edema, dizziness and tinnitus, dry stool, and loose stool. Tongue and pulse conditions refer to red tongue with little coating and thready or weak pulses. Each primary symptom is given a score of 2, 4, or 6; each secondary symptom is given a score of 1, 2, or 3; and the total score is the sum of the scores of the primary and secondary symptoms.

### Interventions

#### Study interventions

The participants will be randomly assigned to five groups at a 1:1:1:1:1 ratio. All of the drugs and dummies will be orally administered for 48 weeks.

-  The SYKFT group will receive SYKFT at 2.4 g three times daily + losartan potassium dummy at two tablets once daily.
-  The losartan potassium 50 mg group will receive SYKFT dummy at five tablets three times daily + losartan potassium at 50 mg once daily + losartan potassium dummy at one tablet once daily.
-  The losartan potassium 100 mg group will receive SYKFT dummy at five tablets three times daily + losartan potassium at 100 mg once daily.
-  The SYKFT + losartan potassium 50 mg group will receive SYKFT at 2.4 g three times daily + losartan potassium at 50 mg once daily + losartan potassium dummy at one tablet once daily.
-  The SYKFT + losartan potassium 100 mg group will receive SYKFT at 2.4 g three times daily + losartan potassium at 100 mg once daily.

#### Concomitant medications

For patients whose blood pressure increases during the follow-up, neither ACEI nor ARB can be used; thus, calcium antagonists, α- and β-receptor blockers, and diuretics (excluding spironolactone) can be applied to control blood pressure to <140/90 mmHg. Non-study antihypertensive drugs will be adjusted in a timely manner if blood pressure decreases, even reaching <90/60 mmHg, during the follow-up. Patients who develop hyperlipidemia, infection, edema, or hypercoagulability will be symptomatically treated. Glucocorticoids, immunosuppressants, or *Tripterygium* drugs will be forbidden throughout the entire study process. Throughout the study, the investigators will record the use of all concomitant medications in detail on a case report form (CRF).

#### Outcome measures

The primary efficacy outcome will be the post-treatment change in the 24-hour proteinuria level, and the secondary efficacy outcomes will be the post-treatment changes in the serum creatinine level, estimated glomerular filtration rate, traditional Chinese medicine syndrome score, and serum albumin level. Outcome measurements will be performed at weeks 0, 4, 8, 12, 24, 36, and 48 after enrollment.

In addition to measurement of the primary and secondary efficacy outcomes, the following measurements will be performed at each follow-up visit: routine blood tests (red blood cell count, hemoglobin level, platelet count, white blood cell count, and white blood cell differential) and blood biochemical examination (levels of fasting blood glucose, serum creatinine, blood urea nitrogen, uric acid, alanine aminotransferase, aspartate aminotransferase, total protein, albumin, total cholesterol, triglyceride, low-density lipoprotein, and serum potassium). Furthermore, the levels of C-reactive protein, IgA, IgG, IgM, interleukin-6, and interleukin-8 will be measured at weeks 0, 24, and 48.

Safety indicators will include a reduction in the white blood cell count, liver function abnormalities, hyperkalemia, and other adverse events.

#### Quality control and quality assurance

A Clinical Trial Standard Operating Procedures training conference was conducted in Beijing to ensure that each site conducts the study according to the Standard Operating Procedures. All investigators from each site attended the conference and received training.

The monitoring procedures will be conducted at every site by the contract research organization throughout the study. The investigator is responsible for providing all source documents, including the CRFs and other study-related files. Confidentiality agreements for each participant will be implemented according to local regulations. The investigator will periodically monitor the CRFs according to a monitoring plan to ensure completeness of the study and validity and accuracy of the data. After verifying the correctness of the research data and original records, these data will be simultaneously entered into the CRFs and input into an electronic data capture system. The electronic data capture system allows only users with specific authority to handle the data; thus, error can be avoided.

At each study site, blood and urine samples will be collected at weeks 0, 24, and 48. After being processed and frozen, the samples will be transported to the central laboratory for unified measurement to reduce data bias.

#### Adverse events

An adverse event will be defined as any untoward medical occurrence that may present itself during the conduct of the study and that may or may not have a causal relationship with the study procedures. A serious adverse event will be defined as an adverse event resulting in death, a life-threatening adverse experience, hospitalization, or significant disability/incapacity [36].

SYKFT has been used for many years and no obvious adverse reactions have been observed or reported. Losartan potassium is well tolerated; its potential adverse reactions are mild and transient and include dizziness, dose-related orthostatic hypotension with an incidence of <1%, and hyperkalemia (serum potassium level of >5.5 mmol/L) with an incidence of 1.5%. All adverse events will be recorded in detail using medical terminology throughout the study. Severe adverse events will be reported to the institutional review board and/or independent ethics committee and the principal investigator within 24 hours.

#### Withdrawal

Withdrawal criteria have been established to ensure participant safety during the study. Withdrawal criteria include patient exhibition of a state that is inappropriate for continuation in the trial, violation of the protocol during the trial, the desire to withdraw from the study at the patient’s own will, or a decision by the ethics committee or sponsor to terminate the study. Participants who meet the withdrawal criteria will be withdrawn from the study and not replaced.

#### Randomization and allocation

Independent biostatisticians with no relationship to the data management and data statistical analysis team will use the SAS 9.1.3 software (version 9.1.3; SAS Institute, Inc., Cary, NC, USA) to generate random numbers according to the block randomization method. This will allow them to allocate the random numbers into five groups at 1:1:1:1:1 ratio. The investigators will allocate the random numbers to eligible participants according to the enrollment sequence. Each site must recruit at least 10 eligible participants initially, and subsequently recruit participants according to the study pace; random numbers and the matching drugs will be complemented by block size. All trial drugs will be dispensed and reclaimed by an independent drug administrator at each site; this administrator will also be responsible for storing and recording the trial drugs.

#### Blinding

The study will have a double-blind design; the participants, investigators, and statisticians will all be blind to the group allocation. We will produce both the SYKFT and losartan potassium dummy agents, which will be identical to the real medications in shape, size, taste, weight, and color. Moreover, the packages for both the dummy agents and real drugs will be identical. Drug packaging will be executed by the personnel responsible for blind coding and personnel from the sponsor unit, neither of whom will have a relationship with the trial. The block size and initial value seed for randomization will be sealed in the blind codes as confidential data. After blind coding, the bind-coding personnel will hand over the sealed blind code to the sponsor unit, principal investigator, and statistician responsible for statistical analysis of the data, respectively.

Each participant will be given an opaque emergency envelope containing the information on the participant’s random number and the matching treatment group. This emergency envelope can be used for emergency unblinding when an investigator considers that revealing the trial drug is needed to handle an emergency event or adverse event.

#### Sample size

Based on the study-related literature, experts’ opinions, and previous study results [28, 30, 31, 37–39], the following will be assumed: the average post-treatment changes in the 24-hour proteinuria level in the SYKFT group, losartan potassium 50 mg group, losartan potassium 100 mg group, SYKFT + losartan potassium 50 mg group, and SYKFT + losartan potassium 100 mg group will be 1.00, 1.00, 1.05, 1.08, and 1.10 g per 24 hours, respectively. The standard deviation will be 0.2 g in every group, the significance level will be α = 0.05, and the power will be (1-β) = 0.9. Adjusting for 20% loss, we will need a sample size of 144 participants in each group. Thus, the total number of participants will be 720.

#### Statistical analysis

The principal analysis will be performed according to the intent-to-treat principle in a full analysis set comprising participants who have been randomly allocated into groups and have taken at least one dose of the study drugs. The set will be obtained after the minimal and reasonable removing method has been used among all randomized participants. Missing data will be adjusted using the last observation carried forward method. Patients who complete the study and comply well with the study protocol without major protocol violations will constitute the per-protocol set, and good compliance will be defined as the fact that the dose of the drug actually taken equals 80% to 120% of the required dose. All participants who have taken at least one dose of the study drugs will be included in the safety analysis set. The full analysis set will be used as the primary analysis population for efficacy analysis, while the per-protocol set will be used as the secondary analysis population for efficacy analysis. The safety analysis set will be used for safety analysis. Detailed classification criteria for the data sets will be determined and be kept on record by a data verification meeting before the database is locked.

Frequency tables, percentages, or constituent ratios will be used to describe the categorical data. Mean and standard deviation or median, first quartile, and third quartile will be used to describe continuous data. Categorical data will be analyzed by the chi-squared test, Fisher’s exact test, the Wilcoxon rank sum test, and the CMHX2 test. Continuous data in each group will be described by mean ± standard deviation and will undergo analysis of variance followed by pairwise comparison. The Wilcoxon rank sum test or Wilcoxon signed rank test will be used if the data exhibit an abnormal distribution. Covariance analysis will be used to assess factors that may affect the efficacy outcomes when appropriate. All statistical analyses will be run using SAS 9.1.3 software (version 9.1.3; SAS Institute, Inc., Cary, NC, USA). Hypothesis testing will uniformly involve a two-tailed test, and both the test statistic quantity and corresponding *P* value will be calculated. Statistical significance will be declared if the *P* value is <0.05.

## Discussion

Primary glomerulonephritis is a major cause of CKD [40–42]. Effective control of proteinuria and protection of kidney function play key roles for the treatment of CKD. Previous research has demonstrated that SYKFT provides a greater advantage in improving the clinical symptoms of CKD, controlling proteinuria, protecting kidney function, and reducing the side effects of Western medicine [38, 39]. SYKFT includes Chinese medicinal herbs with synergic effects, demonstrate stable long-term efficacy, have no toxicity or side effects after many years of use, and exhibit good treatment compliance among patients. However, there is a lack of data derived from randomized controlled trials on the use of SYKFT. We have designed the present multicenter, prospective, double-blind, double-dummy, randomized controlled clinical trial among various sites distributed in 19 provinces and municipalities. An important feature of this study is that participants have a wide geographical distribution and many participating sites are included; thus, poor generalization resulting from a narrow geographical distribution and single center can be avoided, allowing for relatively good generalization and applicability.

The results of this trial will provide solid evidence for evaluation of the efficacy and safety of SYKFT versus losartan potassium in controlling proteinuria in patients with primary glomerulonephritis. Moreover, we hypothesize that SYKFT + losartan potassium therapy can decrease the level of proteinuria in patients with primary glomerulonephritis to a larger extent than SYKFT or losartan potassium therapy alone.

## Trial status

Recruitment commenced in April 2014. Both the recruitment and follow-up of patients are currently in progress. Completion of recruitment is expected in December 2014.

## Electronic supplementary material

Additional file 1: Institutional Review Board/Ethics committee name and approval numbers.(DOCX 21 KB)

Below are the links to the authors’ original submitted files for images.

Authors’ original file for figure 1

## Abbreviations

ACEI: angiotensin converting enzyme inhibitors
ARB: angiotensin II receptor blockers
CKD: chronic kidney disease
CRF: case report form
SYKFT: Shenyankangfu tablets.
