# Supplementary material for: Efficacy and safety of Shenyankangfu tablets for primary glomerulonephritis: study protocol for a randomized controlled trial
Source: Trials. 2014 Dec 5;15:479. doi: 10.1186/1745-6215-15-479 (PMC4289030; doi:10.1186/1745-6215-15-479)
Supplement: Supplementary file 1 — Additional file 1: Institutional Review Board/Ethics committee name and approval numbers.(DOCX 21 KB) [file 13063_2014_2340_MOESM1_ESM.docx]

| **Additional file 1.** **Ethics committees /Institutional Review Board name and approval numbers** | |
| --- | --- |
| Ethics committees /Institutional Review Board name | Approval number |
| Medical Ethics Committee of the Chinese PLA General Hospital | S2013-055-03 |
| Ethics Committee of Guang’ anmen Hospital, China Academy of Chinese Medical Sciences | 2013EC099-01 |
| Medical Ethics Committee of Xiyuan Hospital CACMS | 2013XL044-3 |
| Medical Ethics Committee of Beijing Friendship, Capital Medical University | BJFH-EC/2013-070 |
| Ethics Committee of General Hospital of Chengdu Military Region of PLA | 2013-19 |
| Sichuan Regional Ethics Review Committee on Traditional Chinese Medicine | 2013KZL-014 |
| Medical Ethics Committee of First Affiliated Hospital of Dalian Medical University | 2014-13 |
| Institutional Ethics Committee of Guangdong Provincial Hospital of Traditional Chinese Medicine | B2013-168-01 |
| Ethics Committee of Guangzhou First People's Hospital | SOP-CX-011-03 |
| Medical Ethics Committee of Hangzhou Hospital of Traditional Chinese Medicine | 2013LL055 |
| Ethics Committee of The Third Hospital of Hebei Medical University | 2013-004-2 |
| Institutional Ethics Committee of Henan Provincial People’s Hospital | 2014-018 |
| First Affiliated Hospital of Heilongjiang University of Chinese Medicine | HZYLL203103302 |
| Ethics Committee of The Second Hospital of Jilin University | 2013-063 |
| Ethics Committee of Jiangsu Province Hospital | 2013-MD-171.A1 |
| Medical Ethics Committee of Jiangxi Provincial People’s Hospital | 2013(09) |
| Medical Ethics Committee of Medical Ethics Committee of Beidaihe Sanatorium of Beijing Military Mrca | 2013-10-25 |
| Ethics Committee of Daping Hospital, Research Institute of Surgery Third Military Medical University | （2013）-028-01 |
| Ethics Committee of Shanxi Hospital of Integrated Traditional and Western Medicine | 2013001 |
| Ethics Committee of Second Hospital of Shanxi Medical University | [2014]YW-012 |
| Ethics Committee of First Hospital of Shanxi Medical University | 2013-Y9 |
| Ethics Committee of Shaanxi Traditional Chinese Medicine Hospital | 2013- 11 |
| Ethics Committee of Shanghai Sixth People’s Hospital | 2013-58-（1） |
| Shanghai Jiaotong University School of Medicine, Renji Hospital Ethics Committee | [2014]4 |
| IRB of Shuguang Hospital Affiliated With Shanghai University Of Traditional Chinese medicine | 2013-304-73-01 |
| Ethics Committee of Xinhua Hospital Affiliated to Shanghai Jiaotong University School of Medicine | XHEC-A-2014-003 |
| Shanghai Changhai Hospital Ethics Committee | CHEC2014-033 |
| Ethics Committee of Sichuan Provincial Academy of Medical Sciences, Sichuan Provincial People’s Hospital | 2013-45 |
| IEC of Institution For National Drug Clinical Trials, Tangdu Hospital, Fourth Military Medical University | 2014017 |
| IEC of The First Affiliated Hospital of Tianjin University of Traditional Chinese Medicine | TYLL2013[E]036 |
| Drug Ethics Committee of Tianjin Medical University General Hospital | IRB2013-069-03 |
| Medical Ethics Committee of The First Affiliated Hospital Of Xi’an Jiaotong University | 2013-51-1 |
| Ethics Committee of Xijing hospital, First Affiliated Hospital of The Fourth Military Medical University | 2013-09-24 |
| Ethics Committee of The Second Xiangya Hospital of Central South University | 2013-99 |
| Ethics Committee of The Third Xiangya Hospital of Central South University | 14017 |
| Ethics Committee of Xiangya Hospital of Centre South University | 201403011 |
| Medical Ethics Committee of Tongde Hospital of Zhejiang Province | [2014]005 |
| Ethics Committee of Zhejiang Provincial People’s Hospital | 2013YW016 |
