# Supplementary figures and images for: Efficacy and safety of Shenyankangfu tablets for primary glomerulonephritis: study protocol for a randomized controlled trial
Source: Trials. 2014 Dec 5;15:479. doi: 10.1186/1745-6215-15-479 (PMC4289030; doi:10.1186/1745-6215-15-479)

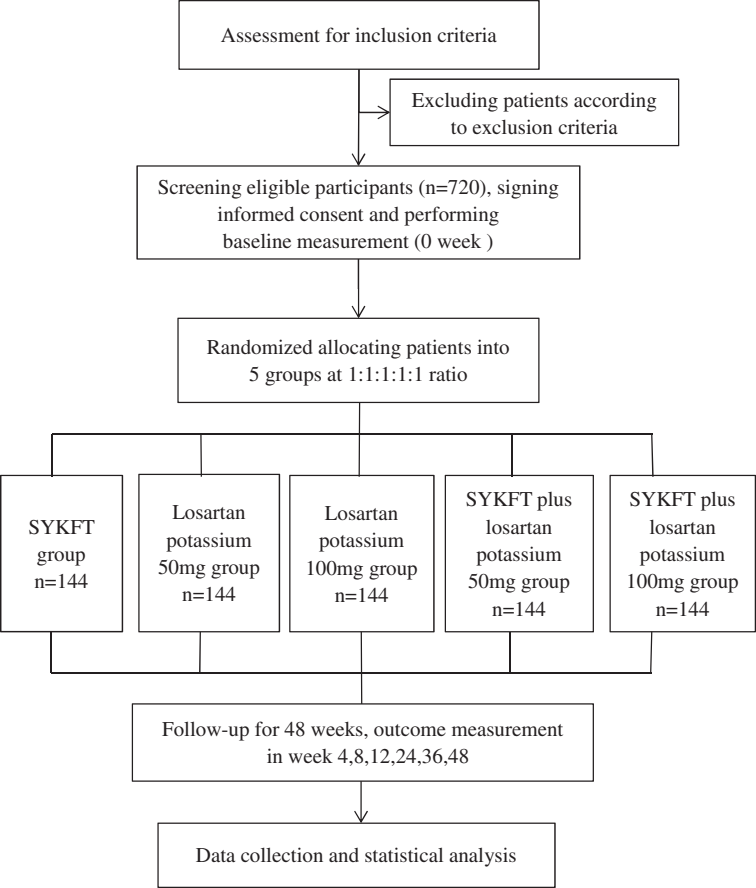

Supplement: Supplementary file 2 — Authors’ original file for figure 1 [file 13063_2014_2340_MOESM2_ESM.pdf]
